# Association Between Vitamin D Deficiency and Autoimmune Thyroid Disorder: A Systematic Review

**DOI:** 10.7759/cureus.25869

**Published:** 2022-06-12

**Authors:** Sabah A Khozam, Abdulhadi M Sumaili, Mohammed A Alflan, Rawan As'ad Salameh Shawabkeh

**Affiliations:** 1 Department of Internal Medicine, Assir Central Hospital, Abha, SAU; 2 Department of Internal Medicine, Armed Forces Hospital - Southern Region, Khamis Mushait, SAU; 3 Department of Endocrinology, Armed Forces Hospital - Southern Region, Khamis Mushait, SAU

**Keywords:** autoimmune thyroid disorder, hashimoto thyroiditis, graves’ disease, 25(oh)d, vitamin d

## Abstract

Despite recent evidence that low serum 25-hydroxyvitamin D (25(OH)D) levels and deflects may influence the emergence of autoimmune thyroid disorders (AITD), the relationship between vitamin D deficiency and Graves’ disease (GD) and Hashimoto's thyroiditis (HT), which comprise AITD, remains unclear. We retrieved studies that described vitamin D association with HT and GD from PubMed/Medline, Google Scholar, and the Cochrane Library. We included research studies that compared vitamin D levels and deficiency or sufficiency between AITD cases such as HT and GD cases and control subjects. The final assessment comprised 11 studies that recruited 1952 AITD cases (HT and GD) that were published between 2011 and 2021; these were included in the final review. All the included studies were observational, and more precisely, case-control studies that recruited healthy subjects as well as controls. The majority of the studies reviewed indicated that HT and GD patients have a greater prevalence of vitamin D deficiency or low serum 25 (OH)-D levels. Two studies failed to establish an association between vitamin D deficiency and HT and GD disease. In conclusion, vitamin D deficiency or insufficiency can increase the rate of autoimmune diseases such as HT and GD. Randomized controlled trials with a longer follow-up period are needed to confirm the causal relationship between autoimmune thyroid disorder and vitamin D and to provide more reliable insights into the relevance of treatment effects of vitamin D therapy or supplementation.

## Introduction and background

Vitamin D, popularly known as "the Sun Vitamin," is a steroid with hormone-like properties. It is required for growth and development because it controls the functions of approximately 200 genes [1]. Vitamin D deficiency is an ignored epidemic worldwide [1]. Vitamin D deficiencies have been linked to a higher risk of common malignancies, hypertension, infectious diseases, and autoimmune disease [2-3]. The relevance of vitamin D as an immune regulator has been highlighted in recent years. The immune system (B and T cells as well as antigen-presenting cells) can generate the active compound of vitamin D, which has immunomodulatory effects, because of the expression of 1-hydroxylase (CYP27B1) in their cells [4]. Furthermore, the appearance of the vitamin D receptor in these cells shows that vitamin D has a local effect on the immunological system. The relationship between vitamin D receptor or CYP27B1 gene polymorphisms and the pathophysiology of various autoimmune disorders backs up these findings [4]. Vitamin D has been discovered to play a key role in immune system function, including adaptive and innate immunity [5]. The immune system, including B and T cells, can generate the active compound of vitamin D, which has immunomodulatory effects [5]. Vitamin D deficiency has already been related to a range of disorders, and a sufficient level of vitamin D is essential for human health. According to the Endocrine Society Guidelines (ECG), sufficiency is defined as 25(OH)D serum levels equal to or greater than 30 ng/mL and up to 100 ng/mL (75 nmol/L up to 250 nmol/L), insufficiency is defined as 25(OH)D serum levels between 20 and 29.9 ng/mL, and deficiency is defined as serum 25(OH)D levels less than 20 ng/mL [6].

The most prevalent organ-specific autoimmune diseases are autoimmune thyroid disease (AITD), which affects 2% to 5% of the community [7]. AITDs are caused by an immune system imbalance, which results in an immunological reaction to the thyroid gland. Hashimoto's thyroiditis and Graves' disease are the two most common clinical manifestations of AITD [8], and hypothyroidism and hyperthyroidism are caused by these factors, respectively [9]. Several recent research studies have concluded a relationship between vitamin D insufficiency or deficiency and autoimmune thyroid illnesses like Hashimoto's thyroiditis (HT) and Graves’ disease (GD) [10].

Our systematic review focuses on the reported association between vitamin D status and autoimmune thyroid disorders such as GD and HT. The purpose of this study was to support the association between HT and GD with vitamin D deficiency by collecting data from studies that included control subjects.

## Review

Methodology

Definition of Outcomes and Inclusion Criteria

We aimed to investigate the association between vitamin D deficiency and autoimmune thyroid disorders. These conditions include Hashimoto’s thyroid (HT) disorder and Graves' disease (GD). Consequently, we included original investigations that recruited a control group for comparing patients with HT and GD. Original studies that described patients with either GD or HT or both were considered the primary inclusion criteria. The review included studies that measured serum 25(OH)D levels and reported vitamin D deficiency/insufficiency in either quantitative or qualitative form. Moreover, we have included studies published between 2011 and 2021. Studies without control groups, case reports with limited sample sizes, and those without descriptive statistics were also excluded from this review. Other exclusion criteria were animal studies, consisting of duplicate data, not related to AITD, did not contain vitamin D information, non-original investigations or incomplete studies, abstract-only articles, protocols, theses, and articles that weren’t published in English or with no available English information.

Search Strategy

Relevant literature was searched in multiple databases, including PubMed/Medline, Google Scholar, and the Cochrane Library. The following specific keywords were used alone or in combination for the search: vitamin D, 25-hydroxyvitamin D, autoimmunity, autoimmune thyroid disorders, Hashimoto’s thyroiditis, and Graves’ disease. Boolean operators (AND, OR, NOT) were also used to increase the sensitivity of the search. Our search strategy was limited to the title and abstract of the search results to utilize all the relevant studies only. All of these results were exported to an Endnote library to identify and execute all duplicates between the different searched databases. Furthermore, we manually searched all similar article sections in PubMed and included studies and relevant reviews for possible detection of any missed studies by the main electronic search strategy. The Preferred Reporting Items for Systematic Reviews and Meta-Analyses (PRISMA) criteria were followed in the present systematic review.

Quality Assessment

We utilized the modified Newcastle-Ottawa scale (NOS) for case-control studies [11], which mainly contains four domains, including the quality of methods, compatibility, assessment, and reporting of the outcomes. Studies were graded from 0 to 10 based on the degree of bias. If the study received a score of ≥ 7 on the Cochrane's Newcastle-Ottawa Scale evaluation standard for case-control studies, it was rated a high-quality study.

Results

Search Results

**Figure 1:**
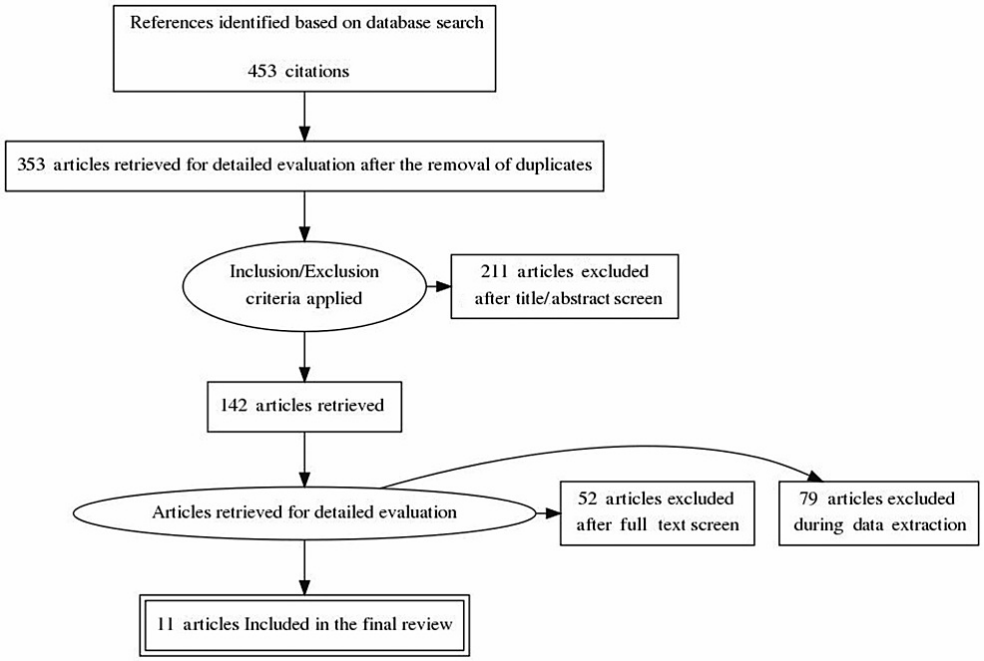
PRISMA figure for the extracted articles PRISMA: Preferred Reporting Items for Systematic Reviews and Meta-Analyses

By conducting the search strategies, we managed to find a total of 462 citations that were then shortened to 353 after the removal of duplicates. Following title and abstract screening, only 142 citations were eligible for the next steps. Full-text screening showed that only 11 articles matched our inclusion and exclusion criteria. The detailed search strategy and screening are shown in Figure 1.

Results of the Quality Assessment

Our assessment of bias for the included studies showed that most of these studies had good quality and less risk of bias, while the rest of them had only excellent and satisfactory results. None of the included studies showed unsatisfactory results.

Characteristics of the Studies Included

We examined 11 studies published between 2011 and 2021 that enrolled 9967 subjects; 1952 cases and 8015 controls. All of the included studies were observational studies, including case groups. Regarding the countries of the included studies, China and Turkey were countries with two studies each while Italy, Tuzla, Brazil, Sweden, Japan, Turkey, Saudi Arabia, and Hungary were all represented by a single study. All the baseline characteristics of the study included in the review are shown in Table 1.

**Table 1:** Baseline characteristics of the included studies in this review AITD: autoimmune thyroid disorders, HT: Hashimoto's thyroiditis, GD: Graves disease

Reference	Year	Country	design	Sample size	case	control present	AITD
Chao et al. [12]	2020	China	Observational study	5262	373	Yes	HT
Cvek et al. [13]	2021	Italy	Observational study	637	461	Yes	HT
Sulejmanovic et al. [14]	2020	Tuzla	Observational study	150	50	Yes	Autoimmune Hypothyroidism
Botelho et al. [15]	2018	Brazil	Observational study	159	88	Yes	HT
Planck et al. [16]	2018	Sweden	Case-control	2597	292	Yes	GD
Ma et al. [17]	2015	China	Case-control	210	140	Yes	HT, GD
Unal et al. [18]	2014	Turkey	Case-control	405	281	Yes	HT, GD
Mackawy et al. [19]	2013	Saudi Arabia	Case-control	60	30	Yes	HT
Yasuda et al. [20]	2012	Japan	Case-control	72	26	Yes	GD
Kivity et al. [21]	2011	Hungary	Case-control	92	50	Yes	HT, GD
Tamer et al. [22]	2011	Turkey	Case-control	323	161	Yes	HT

Discussion

Vitamin D and Hashimoto’s Thyroiditis

Patients with HT had significantly lower levels of vitamin D as compared to the control groups across the four studies included in this review. We identified 1657 HT patients in our study. Among which, 1108 HT patients showed a significantly lower level of vitamin D. Previous studies in recent years have led to presume an association between vitamin D deficiency and Hashimoto’s thyroid disorders. Chao et al. reported that when compared with a non-HT control group, the HT group presented with a reduced level of 25(OH)D (p = 0.014) [12]. Similarly, Ma et al. found an inverse relationship between serum 25(OH)D and autoimmune thyroid disorders such as HT in comparison to the control subjects (31.00 versus 41.33 nmol/L, P < 0.001), and no difference was found between HT cases and GD cases (p=0.97) [17]. They reported a higher percentage of vitamin D deficiency in case subjects (HT=94.29%, GD=92.86% versus control =77.14%). Hypothyroidism patients had hypocalcemia and hypovitaminosis D according to the findings of Mackawy et al. [19]. They discovered that people with hypothyroidism had considerably lower serum 25(OH)D levels than controls (t= −11.128, P =0.000). These results were confirmed by several recent studies. According to a meta-analysis of 20 articles, vitamin D deficiency is more prominent in AITD patients and these patients have lower serum 25(OH)D levels [23]. These findings indicate that a lack of vitamin D may have an impact on AITD pathogenesis. The results from a case-control study conducted by Sulejmanovic et al. confirm the findings from the aforementioned studies that had significantly lower levels of vitamin D in AITD patients [14]. This result was confirmed by Tamer et al. [22]. They reported that vitamin D insufficiency is associated with HT cases (148 of 161, 92%). The rate of vitamin D deficiency in these HT cases tended to be higher in patients having overt hypothyroidism (94%) and subclinical hypothyroidism (98%) (Table 2).

**Table 2:** Summary of the outcomes of the included studies in this review a: deficiency, b: insufficiency, NR: not reported, NS: not significant, *Significant p-value (<0.05), GD: Grave’s disease, HT: Hashimoto’s disease, AITD: autoimmune thyroid disorder

Author	GD	HT	AITD	Control group	D-25(OH) levels		P-value	Vitamin D deficiency/sufficiency	Findings
					case	control			
Chao et al. [12]	NR	373	NR	4889 non-HT	15.81±6.42	16.66±6.51	0.014*	<20 ng/ml^a^	72.0% non - HT group and 76.1% HT group, (*p* = 0.238).
Cvek et al. [13]	NR	461	NR	176 non-HT	NR	NR	NR	<20 ng/Ml^a^	NS
Sulejmanovic et al. [14]	NR	NR	50	50 non-AITD and 50 healthy subjects	20.76±6.31	24.37±9.05	<0.001*	20 ng/ml^a^	68% in AITD and 24% in control
Botelho et al. [15]	NR	88	NR	71 non-HT subjects	26.4 (7.6–48.2)	28.6 (13–51.2)	0.1917	<30 ng/dl^b^	71.8% in HT, 59.1% in control (p=0.1024)
Planck et al. [16]	292	NR	NR	2305 normal subjects	55.0 ± 23.2	87.2 ± 27.6	<0.001*	≤25 nmol/L^a^	Vitamin D deficiency and insufficiency were higher in cases than in controls
Ma al. [17]	70	70	NR	70 healthy subjects	GD = 31.71± 13.10, HT= 31 ±11.15	41.33 + 14.48	<0.001*	<50 nmol/L^a^	65% in GD, 66% in HT, and 54% in controls, p<0.002*
Unal et al. [18]	27	254	NS	124 healthy subjects	HT =17.05 (5.4-80), GD=14.9 (4-3.9)	19.9 (9-122.7)	<0.001*	<20 ng/ml^a^	63% in HT and 85.2% in GD
Mackawy et al. [19]	NR	30	NR	30 healthy subjects	14.79 ± 2.11	44.53 ± 14.91	<0.001*	NR	NR
Yasuda et al. [20]	26	NR	NR	46 healthy subjects	14.4 ± 4.9	17.1 ± 4.1	<0.05*	<15ng/ml^b^	65.4% in GD. 32.4% in control (*P* < 0.05)
Kivity et al. [21]	28	22	NR	42 non-AITD	NR	NR	NR	<10ng/ml^a^	79% HT, 64% in GD,30% in control, (P<0.001*)
Tamer et al. [22]	NR	161	NR	162 healthy subjects	16.3 ± 10.4	29.6 ± 25.5	<0.001*	<30ng/ml^b^	insufficiency 92% in cases and 63% in controls (p<0.001*)

Two studies in our review failed to establish an association between serum vitamin D levels or vitamin D deficiency with HT subjects. When HT case groups of all stages of disease (MILD and OVERT) were compared to control groups, Cvek et al. found no statistical differences in serum levels of 25(OH) D or the prevalence of its deficiency [13]; however, overt hypothyroidism may be associated with a gradual decrease in the patients' serum vitamin D levels. A study conducted by Botelho et al. also reported that lower levels of vitamin D have not been linked to HT cases, but thyroxin levels were identified as a contributing factor to vitamin D deficiency [15]. Both studies had flaws, such as a reduced number of controls and one study's inability to reach the recommended sample size, as well as a sample collection time that spanned the spring and summer. It also has an impact on the outcome. 

Numerous studies have found a link between vitamin D insufficiency and HT, and vitamin D therapy or supplementation may be indicated for HT patients [24-25]. A growing body of research suggests that vitamin D receptor (VDR) polymorphisms are linked to an increased risk of AITD [26-27]. Even if the VDR locus does not appear to have a role in HT susceptibility, vitamin D insufficiency may play a role in illness initiation by functioning as an environmental trigger. Several cofactors, such as sun exposure, obesity, and sedentary behavior, may influence epidemiologic research results, leading to contradictory findings. However, there is rising evidence of a relationship between lower vitamin D levels or deficiency and AITD.

Vitamin D and Graves’ Disease

Our review includes five studies that comprise Graves' disease patients as case subjects. Among the total cases, 443 patients were GD patients. Clinical and experimental evidence suggests that GD is caused by the complicated interaction of genetic and ecological factors that culminate in the lack of immunological tolerance to thyroid antigens and the initiation of an immune response [28-29]. Vitamin D is frequently mentioned as one of the environmental elements involved in the immunopathogenesis of AITD [30]. A meta-analysis including 27 studies conducted by Xu et al. confirms that vitamin D deficiency may increase the risk of Graves’ disease [31]. Patients with GD, in particular, were more likely than controls to be vitamin D deficient (OR = 2.24, 95% CI: 1.31-3.81). The majority of existing information indicates that patients with Graves' disease have an increased prevalence of low serum vitamin D levels and deficiencies [20,32-33].

Planck et al. compared the cases with a large group of controls and explained that vitamin D levels were observed to be considerably lower in GD patients compared to controls [16]. However, there was no relationship between serum vitamin D levels and a variety of laboratory and clinical findings in GD. In a case-control study, Unal et al. found that GD cases had lower 25(OH)D than healthy subjects and HT cases [18]. Based on their determined cut of values, 60.7% of the population had vitamin insufficiency, which was higher in patients with GD (85.2%). Serum 25(OH) D3 levels in GD cases were statistically lower than controls (14.4 ± 4.9 vs. 17.1 ± 4.1 ng/ml, P= 0.05), as was vitamin D deficiency (65.4 vs. 32.4%, P= 0.05), according to Yasuda et al. [20].

In a report by Kivity et al., the percentage of vitamin D deficiency was considerably greater in AITD patients than in 98 healthy controls (72% vs. 30.6%, respectively, p=<0.001) [21]. However, in a sub-analysis, they discovered no significant relationship was observed in 25(OH) D serum levels and vitamin D deficiency in GD cases that appeared to be similar to the control cases, whereas mild and treated HT patients showed significant differences in vitamin D serum levels and deficiency when contrasted to the control subjects. This might be due to the smaller sample size, heterogeneity of the sample population, and social behavior of the included cases.

Several studies have concluded Graves' disease patients have a greater prevalence of vitamin D deficiency or low serum 25-OH-D levels [20,31]. Based on the outcomes of the included study, we verified that vitamin D deficiency may raise the risk of Graves' illness. Nevertheless, it has to be seen through more experimental studies whether vitamin D deficiency promotes illness onset or whether its supplementation has any therapeutic value in Graves' disease.

It's worth noting that the study that found the relationship between low serum vitamin D levels and autoimmune thyroid disorders was based on observational research, which could be skewed. Some studies reported a lower number of study populations and heterogeneity between them. Our study's strength was that we employed a diverse set of references to assess the relationship between AITD and vitamin D, which minimized publication bias.

## Conclusions

Vitamin D deficiency or insufficiency can increase the rate of autoimmune diseases like HT and GD. More randomized controlled trials with a longer follow-up period are needed to confirm the causal relationship between autoimmune thyroid disorder and vitamin D and to provide more reliable insights into the relevance of treatment effects of vitamin D.
